# Prevalence and Risk Factors for Medical Debt and Subsequent Changes in Social Determinants of Health in the US

**DOI:** 10.1001/jamanetworkopen.2022.31898

**Published:** 2022-09-16

**Authors:** David U. Himmelstein, Samuel L. Dickman, Danny McCormick, David H. Bor, Adam Gaffney, Steffie Woolhandler

**Affiliations:** 1 The City University of New York at Hunter College, New York, New York; 2 Department of Medicine, Cambridge Health Alliance, Cambridge, Massachusetts; 3 Harvard Medical School, Boston, Massachusetts; 4 Public Citizen Health Research Group, Washington, DC; 5 Planned Parenthood of Montana, Billings

## Abstract

**Importance:**

Cost barriers discourage many US residents from seeking medical care and many who obtain it experience financial hardship. However, little is known about the association between medical debt and social determinants of health (SDOH).

**Objective:**

To determine the prevalence of and risk factors associated with medical debt and the association of medical debt with subsequent changes in the key SDOH of food and housing security.

**Design, Setting, and Participants:**

Cross-sectional analyses using multivariable logistic regression models controlled for demographic, financial, insurance, and health-related factors, and prospective cohort analyses assessing changes over time using the 2018, 2019, and 2020 Surveys of Income and Program Participation. Participants were nationally representative samples of US adults surveyed for 1 to 3 years.

**Exposures:**

Insurance-related and health-related characteristics as risk factors for medical debt; Newly incurred medical debt as a risk factor for deterioration in SDOHs.

**Main Outcomes and Measures:**

Prevalence and amounts of medical debt; 4 SDOHs: inability to pay rent or mortgage or utilities; eviction or foreclosure; and food insecurity.

**Results:**

Among 51 872 adults surveyed regarding 2017, 40 784 regarding 2018 and 43 220 regarding 2019, 51.6% were female, 16.8% Hispanic, 6.0% were non-Hispanic Asian, 11.9% non-Hispanic Black, 62.6% non-Hispanic White, and 2.18% other non-Hispanic. A total of 10.8% (95% CI, 10.6-11.0) of individuals and approximately 18.1% of households carried medical debt. Persons with low and middle incomes had similar rates: 15.3%; (95% CI,14.4-16.2) of uninsured persons had debt, as did 10.5% (95% CI, 10.2-18.8) of the privately-insured. In 2018 the mean medical debt was $21 687/debtor (median $2000 [IQR, $597-$5000]). In cross-sectional analyses, hospitalization, disability, and having private high-deductible, Medicare Advantage, or no coverage were risk factors associated with medical indebtedness; residing in a Medicaid-expansion state was protective (2019 odds ratio [OR], 0.76; 95% CI, 0.70-0.83). Prospective findings were similar, eg, losing insurance coverage between 2017 and 2019 was associated with acquiring medical debt by 2019 (OR, 1.63; 95% CI, 1.23-2.14), as was becoming newly disabled (OR, 2.42; 95% CI, 1.95-3.00) or newly hospitalized (OR, 2.95; 95% CI, 2.40-3.62). Acquiring medical debt between 2017 and 2019 was a risk factor associated with worsening SDOHs, with ORs of 2.20 (95% CI,1.58-3.05) for becoming food insecure; 2.29 (95% CI, 1.73-3.03) for losing ability to pay rent or mortgage; 2.37 (95% CI, 1.75-3.23) for losing ability to pay utilities; and 2.95 (95% CI, 1.38-6.31) for eviction or foreclosure in 2019.

**Conclusions and Relevance:**

In this cross-sectional and cohort study, medical indebtedness was common, even among insured individuals. Acquiring such debt may worsen SDOHs. Expanded and improved health coverage could ameliorate financial distress, and improve housing and food security.

## Key Points

**Question:**

What are the prevalence and risk factors associated with medical debt in the US?

**Findings:**

In this cross-sectional and cohort study of survey data from 2017 to 2019, 10.8% of adults carried medical debt, including 10.5% of the privately insured, and 9.6% of residents of Medicaid-expansion states, significantly fewer than in nonexpansion states. Over 3 years, decreases in health status and coverage loss were significant risk factors associated with acquiring medical debt, which was, in turn associated with a significant 1.7-fold to 3.1-fold higher risk of worsening housing and food security.

**Meaning:**

Results of this study suggest that medical indebtedness is common, even among the insured, and may be associated with subsequent worsening of social determinants of health.

## Teaser

This cross-sectional and cohort study assesses the prevalence of and characteristics associated with medical debt among US adults and evaluates changes in food and housing security among individuals who experience medical debt.

## Introduction

Cost barriers discourage many US residents from seeking medical care and many who obtain it experience financial hardship. Although uninsured individuals are most often harmed, persons with coverage are also at risk. One-fifth of insured adults aged 18 to 64 years incurred unaffordable out-of-pocket costs in 2020, and insurance deductibles left another 7% financially vulnerable.^1^ Among Medicare beneficiaries, out-of-pocket costs averaged $5460 in 2016; half spent at least 12% of their income on such costs, and one-quarter spent at least 23%.^2^

Patients unable to pay medical bills from current income or savings sometimes use credit cards, loans, or mortgages to pay them, negotiate payment plans with health care professionals and hospitals, or fail to pay them. Debt appears to inhibit subsequent care-seeking,^3^ may impair physical and mental health,^4,5^ and may, some suggest, constitute a social determinant of health (SDOH).^6^

Recent studies indicate that delinquent medical bills constitute the majority of all unpaid bills on credit reports.^7,8^ However, those studies' reliance on the limited information available from credit reports precluded analyses of medical debts that are not in arrears or those financed using credit cards, mortgages or loans, debtors’ person-level characteristics, or the downstream consequences of debts. A 2015 survey characterized adults aged 18 to 64 years reporting any medical-bill problems, including bills that were paid on-time but caused hardship, but ascertained little information on medical debts per se.^9^

We assessed the characteristics of adults with medical debt nationally; health-related and insurance-related risk factors for such debt; and the associations of medical debt with food and housing insecurity.

## Methods

The City University of New York and Cambridge Health Alliance institutional review boards do not consider analyses of deidentified public-use data to be human participants research. We followed Strengthening the Reporting of Observational Studies in Epidemiology (STROBE) reporting guideline for cross-sectional studies.

We analyzed data from the Census Bureau 2018, 2019, and 2020 Survey of Income and Program Participation (SIPP). Each year’s SIPP collects data for the previous year from a nationally representative sample of US households. Although the SIPP normally conducts in-person interviews, after March 18, 2020, interviews were conducted only by telephone.

Although the SIPP planned to enroll new panels of respondents each year (and to collect data from each panel for 4 years), a 2019 government shutdown forced SIPP staff to curtail enrollment in the 2019 panel and to abandon plans to follow up that panel. Hence, multiple years of data are available for some respondents initially enrolled in 2018 and resurveyed in the 2019 and 2020 SIPPs, and only 1 year of data are available for persons initially enrolled in 2019 or 2020. In total, our cross-sectional samples included 51 872 respondents to the 2018 SIPP; 47 784 respondents to the 2019 SIPP (including 25 376 who had also provided data in the 2018 SIPP); and 43 330 respondents to the 2020 SIPP (16 836 of whom had also provided data in the 2019 SIPP, and 16 478 of whom had also provided data in both the 2019 and 2018 SIPPs).

The SIPP collects monthly data on income and health insurance, and annual data on demographic characteristics, assets, debts, health-related measures, and 4 SDOHs: (1) food security (high/marginal vs low/very low) based on the standard US Department of Agriculture questions and scale^10^; (2) “unable to pay for rent or mortgage”; (3) “unable to pay utility bills”; and (4) moving due to “eviction or foreclosure”. “Adults” (age ≥15 years) are queried on out-of-pocket spending for medical care and premiums and whether “money was owed for medical bills not paid-in-full during the [year]” and the amount of medical debts, which respondents usually reported in round numbers. SIPP imputes approximately 7% of debt amounts based on respondent-provided ranges (eg, $1000-$2500), and 12% from other data. Dollar figures are top-coded to preserve anonymity.

### Statistical Analysis

We performed cross-sectional analyses on all adults surveyed in each year. Additionally, we performed prospective analyses to assess the longitudinal associations across the years 2018-2019, 2019-2020, and 2018-2020. Statistical significance was defined as a 95% CI excluding 1 for ratios.

#### Cross-sectional Analyses

We tabulated the characteristics of persons with medical debt each year and mean (and median) amounts of such debt. We displayed mean amounts only for 2018 because top-coding differed year to year. We also reported mean out-of-pocket spending for medical care and premiums.

We assessed risk factors for medical indebtedness in 2 sets of multivariable logistic regression models. Because data collection methods varied from year-to-year and some individuals were surveyed in multiple years, we stratified these analyses by year. The first set of stratified models examined health-related risk factors: (1) disability status (yes/no); (2) hospitalization (any/no); or (3) self-reported health status (excellent, very good, good, fair, or poor). Sensitivity analyses examining sick days, hospital days, or doctor visits yielded similar results and are not reported further.

The second set of models assessed insurance-related risk factors for medical debt: (1) type of insurance (private non–high deductible, private high-deductible, Medicare, military, Medicaid or uninsured [eMethods in the Supplement]); (2) type of Medicare coverage among beneficiaries (Medicare Part C [hereinafter, *Medicare Advantage*], traditional [non–Medicare Advantage] Medicare plus private insurance, traditional Medicare without private coverage); and (3) residence in a Medicaid-expansion state.

These models were adjusted for sex (male/female), family income relative to the federal poverty level (<100%, 100%-199%, 200%-299%, 300%-399%, and ≥400%), region (Northeast, Midwest, South, or West), race and ethnicity (Hispanic, non-Hispanic Asian, non-Hispanic Black, non-Hispanic White, and other race and ethnicity), and age (15-39 years, 40-64 years, and ≥65 years). The survey included one question about race and a separate one about Hispanic ethnicity. We classified all persons identifying Hispanic ethnicity as Hispanic and they were excluded from any other category. Age was omitted from models analyzing type of Medicare coverage, and (to avoid colinearity) region from Medicaid-expansion analyses. Analyses of health-related factors included additional adjustment for insurance type.

For each year we then constructed logistic regression models (adjusted for age, income, race and ethnicity, sex and region) to assess medical debt as a risk factor for each SDOH outcome.

#### Prospective Analyses

We used unique household and person identifiers to link respondents across years, and assessed whether adults had newly acquired medical debt (ie, reported medical debt in the later but not the earlier year).

For each pair of years (2017-2018, 2018-2019, and 2017-2019), we constructed logistic regression models with newly acquired medical debt (ie, those reporting debt in the later but not the earlier year) as the outcome and: (1) newly acquired disability; (2) hospitalization in the later (but not earlier) year; (3) loss of health insurance (insured throughout the earlier year but uninsured ≥1 month in the later year); or (4) increased out-of-pocket medical cost as risk factors.

We then constructed logistic regression models assessing newly acquired medical indebtedness as a risk factor for deterioration in each SDOH outcome.

All prospective analyses were adjusted for change in family-income category, as well as baseline age, sex, race and ethnicity, region, and income. Sensitivity analyses that additionally adjusted for educational level (which reduced sample size) yielded closely similar findings and are not reported further.

We explored potential protective effects of insurance coverage among persons hospitalized or newly disabled by repeating those models with added adjustment for whether or not the adult was continuously insured in the periods when they newly acquired debt.

#### Additional Sensitivity Analyses

Because financial shocks other than medical bills (eg, loss of work-related income) could worsen SDOH and contribute to medical debt, we performed sensitivity analyses to explore different specifications of changes in financial status. Two specified income change (relative to federal poverty level) continuously rather than categorically or as a binary indicator of any (vs no) decrease. Two added adjustment for changes in net worth (excluding medical debt) or total nonmedical debts. These sensitivity analyses yielded results closely similar to our main models and are not reported further. Additionally, we examined risk factors for and associations with losing medical debt, which yielded results consistent with our main findings.

In addition, we compared the demographic characteristics and prevalence of medical debt among persons surveyed only once in 2018 to those included in our prospective analyses, and performed prospective analyses examining lagged effects – eg, the association between newly acquiring medical debts 2017-2018 and worsening food security 2017-2019.

We used SAS version 9.4 (SAS Institute Inc) procedures that account for complex sampling and Census Bureau–supplied person-level weights (and replicate weights) appropriate to each analysis’ time frame, which adjust for nonresponse and allow national estimates. Confidence intervals were calculated using the Fay method of balanced repeated replication.

## Results

Table 1 provides the characteristics of the weighted samples: 51.6% were female, 16.8% Hispanic, 6.0% were non-Hispanic Asian, 11.9% non-Hispanic Black, 62.6% non-Hispanic White, and 2.18% other non-Hispanic. Most of the sample had private coverage and 11.2% had below poverty-level family incomes. eTable 1 in the Supplement describes the characteristics of persons with and without medical debt.

**Table 1.** Percent of Adults Carrying Medical Debt During a Year, Average 2017-2019, and Amounts of Debt, 2018

Characteristic	Sample, weighted %^a^	Medical debt 2017-2019, % (95% CI)	Amount of debt, $	
			Medical debt among those with debt 2018, median (IQR)	Mean medical debt among those with debt 2018^b^
Sex				
Male	48.4	9.18 (8.90-9.45)	2000 (600-5000)	32 500
Female	51.6	12.27 (11.96-12.57)	2000 (530-5000)	14 229
Age range, y				
15-39	40.8	9.45 (9.12-9.79)	1570 (583-4000)	16 364
40-64	39.0	13.98 (13.57-14.39)	2000 (700-6000)	27 021
≥65	20.2	7.20 (6.82-7.58)	1250 (350-4000)	15 512
Race and ethnicity				
Hispanic	16.8	10.31 (9.76-10.86)	2000 (600-5000)	19 347
Non-Hispanic Asian	6.0	4.52 (3.92-5.11)	2000 (400-5000)	10 613
Non-Hispanic Black	11.9	16.52 (15.67-17.37)	2000 (600-5000)	12 319
Non-Hispanic White	62.6	10.30 (10.02-10.58)	2000 (500-5000)	25 571
Other^c^	2.8	12.93 (11.66-14.20)	2000 (600-7000)	9336
Marital status				
Married	49.3	10.62 (10.31-10.93)	2000 (500-5000)	24 829
Single, widowed, or divorced	50.7	10.92 (10.63-11.21)	2000 (500-5000)	18 520
Income relative to poverty level				
<100	11.2	12.31 (11.68-12.95)	2000 (700-7350)	20 694
100-199	15.5	15.36 (14.71-16.02)	2000 (600-5000)	20 554
200-299	15.3	14.75 (14.15-15.34)	2000 (600-5000)	19 944
300-399	13.3	12.11 (11.47-12.75)	2000 (517-5000)	14 918
≥400	44.7	7.03 (6.76-7.30)	2000 (500-5000)	28 306
Education				
Less than high school	13.1	14.44 (13.56-15.31)	2000 (700-6000)	23 704
High school	33.9	14.02 (13.53-14.52)	2000 (500-5000)	27 589
Some college	34.5	15.24 (14.73-15.74)	2000 (676-5000)	15 557
Graduate degree	18.5	6.35 (5.87-6.84)	2000 (500-4000)	21 475
Insurance				
Private				
Any type	52.8	10.51 (10.22-18.81)	2000 (600-4900)	17 927
Not high deductible	27.4	9.09 (8.72-9.46)	1500 (500-4000)	15 664
High deductible	25.5	12.04 (11.59-12.49)	2000 (600-5000)	19 673
Medicare (any type)	17.0	9.44 (8.99-9.89)	1900 (450-5000)	24 368
Medicaid	15.0	11.55 (11.01-12.09)	1500 (500-5000)	20 209
Military	5.2	6.85 (6.08-7.63)	1850 (500-4000)	9055
Uninsured	10.0	15.27 (14.38-16.16)	3000 (1000-12 000)	38 311
Type of Medicare coverage				
Medicare Advantage	12.9	10.23 (9.09-11.37)	1500 (450-3400)	17 908
Traditional Medicare plus private	52.6	7.45 (6.96-7.93)	2000 (400-5000)	20 654
Traditional Medicare, no private^d^	34.5	13.06 (12.33-13.79)	1500 (390-5020)	34 210
Health status				
Excellent	25.9	4.86 (4.56-5.17)	1400 (500-3500)	19 099
Very good	31.4	8.05 (7.73-8.37)	1500 (500-4000)	13 308
Good	27.0	13.10 (12.65-13.55)	1800 (569-5000)	18 499
Fair	11.5	20.69 (19.96-21.43)	2200 (742-6000)	24 479
Poor	4.3	24.80 (23.42-26.12)	2800 (800-10 000)	42 687
Disability status				
Disabled	15.6	19.48 (18.89-20.07)	2000 (600-7000)	32 357
Not disabled	84.4	9.15 (8.93-9.38)	2000 (563-5000)	17 278
Hospitalized in past year				
Yes	10.2	22.75 (21.98-23.53)	3000 (1000-9650)	34 291
No	89.8	9.41 (9.19-9.62)	1700 (500-4500)	18 084
No. of doctor visits in year				
0 or 1	40.6	7.74 (7.45-8.04)	1800 (600-5000)	26 480
2 or 3	26.3	9.09 (8.75-9.43)	1500 (500-4000)	13 049
≥4	33.1	15.80 (15.40-16.21)	2000 (600-5000)	22 653
No. of sick days				
None	56.9	7.13 (6.90-7.37)	1300 (500-3610)	19 210
1 to 3	24.2	11.19 (10.78-11.60)	2000 (530-5000)	14 186
>3	18.9	21.16 (20.57-21.76)	2500 (800-6800)	29 078
Food insecurity				
Secure	90.5	9.24 (9.03-9.44)	2000 (500-5000)	19 935
Moderately insecure	5.9	22.84 (21.64-24.04)	2000 (700-5000)	22 805
Very insecure	2.7	29.23 (27.49-30.97)	2000 (700-7000)	32 779
Unable to pay utilities				
Yes	7.4	26.23 (25.09-27.37)	2000 (700-6000)	27 279
No	92.6	9.53 (9.33-9.74)	2000 (500-5000)	20 451
Unable to pay mortgage/rent				
Yes	5.2	25.21 (23.74-26.68)	2000 (750-6000)	26 008
No	94.8	9.99 (9.79-10.19)	2000 (500-5000)	21 089
Moved because of eviction or foreclosure				
Yes	0.6	21.92 (17.90-25.94)	3500 (1000-6000)	31 993
No	99.4	10.71 (10.49-20.92)	2000 (593-5000)	21 554
Region				
Northeast	17.6	8.79 (8.25-9.32)	1500 (500-4000)	19 138
Midwest	20.8	12.13 (11.56-12.70)	2000 (500-5000)	17 942
South	37.8	13.02 (12.61-13.42)	2000 (700-5250)	20 359
West	23.8	7.48 (7.09-7.86)	1730 (500-5000)	33 301
Medicaid expansion state				
Yes	69.4	9.61 (9.35-9.88)	1800 (500-5000)	20 550
No	30.6	13.41 (12.94-13.87)	2000 (700-6000)	23 522
All persons aged >14 y	100	10.77 (10.56-10.99)	2000 (597-5000)	21 687

^a^ Unweighted N = 51 872 in 2017, 40 784 in 2018, and 43 220 in 2019.

^b^ Medical debts were top coded at $963 000.

^c^ Other includes American Indian or Alaska Native alone, Native Hawaiian or Other Pacific Islander alone, and persons reporting 2 or more races.

^d^ Includes persons with Medicare plus Medicaid.

### Cross-sectional

Table 1 also displays the proportion of adults with medical debts, averaged over 3 years, and 2018 debt amounts. eTable 2 in the Supplement provides individual-year data. A total of 10.8%; (95% CI, 10.6-11.0) of adults (and approximately 18.1% of households) had medical debt, a proportion that declined slightly between 2017 and 2019. Among persons who provided data in all 3 years, 19.75%; (95% CI, 19.01-20.48) reported medical debt in at least 1 year.

Among adults with any medical debt, the median amount overall, and for most subgroups was approximately $2000. Mean amounts (2018) were much higher–$21 687 overall (median $2000 [IQR, $597-$5000])–reflecting the very large debts of a small percentage of debtors. Across the entire population, mean medical debt was $2306 per US adult and approximately $4671 per US household. Although median debts were similar in other years, mean amounts were lower, reflecting, at least partly, lower top-coding thresholds.

Persons in the highest educational level and income categories had the lowest rates of medical debt; however, rates differed little between the lowest and middle categories; 12.3% (95% CI, 11.7-13.0) of adults with incomes below poverty level carried medical debt, as did 12.1% (95% CI, 11.5-12.8) of those with incomes 300%-399% of federal poverty level. Women were more likely to have medical debt (12.3%; 95% CI, 12.0-12.6) than men (9.2%; 95% CI, 8.9-9.5). Among racial/ethnic groups, non-Hispanic Black adults had the highest incidence of medical indebtedness (16.5%; 95% CI, 157-17.4); non-Hispanic Asian adults had the lowest (4.5%; 95% CI, 3.9-5.1). All measures of medical need (eg, self-reported health) were associated with medical indebtedness. Medical indebtedness was consistently more frequent among individuals with food or housing insecurity (eg, 25.2% [95% CI, 23.7-26.7] of those unable to pay rent or mortgage).

Uninsured persons had the highest prevalence of medical debt (15.3%; 95% CI, 14.4-16.2) followed by those with high-deductible private plans (12.0%; 95% CI, 11.6-12.5), and those with military coverage (6.9%; 95% CI, 6.1-7.6) or traditional Medicare plus private coverage (7.5%; 95% CI, 7.0-7.9) had the lowest prevalence. Residents of states that had refused the ACA Medicaid expansion were 40% more likely to have medical debts than those in Medicaid-expansion states.

Adults with medical debt reported approximately twice as much out-of-pocket medical spending as others: means of $1485 vs $823 in 2017; $1550 vs $767 in 2018; and $1593 vs $818 in 2019. Medical debtors also paid modestly more toward premiums: $1698 vs $1429 in 2017, $1592 vs $1292 in 2018, and $1671 vs $1337 in 2019.

Medical indebtedness was associated with indicators of medical need after adjustment for other factors, including insurance (Table 2). For instance, poor (vs excellent) self-rated health was associated with approximately 7-fold higher odds of having medical debt, and hospitalization with 3-fold higher odds.

**Table 2.** Cross-sectional Risk Factors for Medical Debt, Stratified by Year and Adjusted

Risk factor	Having medical debt, aOR (95% CI)		
	2017^a^	2018^b^	2019^c^
Health-related risk factors^d^			
Hospitalized during year	2.97 (2.74-3.22)	3.06 (2.79-3.36)	2.94 (2.68-3.22)
Disabled	2.68 (2.48-2.91)	2.45 (2.21-2.70)	2.59 (2.36-2.84)
Health status			
Excellent	1 [Reference]	1 [Reference]	1 [Reference]
Very good	1.778 (1.60-1.98)	1.80 (1.55-2.09)	1.63 (1.42-1.86)
Good	3.01 (2.77-3.43)	2.97 (2.59-3.40)	2.95 (2.60-3.35)
Fair	5.47 (4.86-6.16)	5.85 (5.06-6.77)	5.00 (4.31-5.82)
Poor	7.74 (6.73-8.90)	7.81 (6.45-9.46)	6.82 (5.77-8.05)
Insurance type^e^			
Private			
Not high deductible	1 [Reference]	1 [Reference]	1 [Reference]
High deductible	1.30 (1.19-1.43)	1.41 (1.27-1.58)	1.45 (1.32-1.61)
Medicare	0.84 (0.74-0.99)	0.95 (0.80-1.13)	0.86 (0.73-1.03)
Military	0.42 (0.33-0.52)	0.65 (0.52-0.81)	0.62 (0.50-0.78)
Medicaid	0.59 (0.52-0.66)	0.66 (0.57-0.77)	0.59 (0.51-0.69)
Uninsured	1.31 (1.17-1.46)	1.28 (1.09-1.50)	1.26 (1.08-1.46)
Medicare type^f^			
Traditional Medicare plus private	1 [Reference]	1 [Reference]	1 [Reference]
Medicare Advantage	1.34 (1.06-1.68)	1.33 (1.05-1.67)	1.21 (0.67-1.51)
Traditional Medicare, no private	1.23 (1.06-1.42)	1.15 (0.94-1.40)	1.09 (0.91-1.30)
Medicaid expansion^g^			
Nonexpansion state	1 [Reference]	1 [Reference]	1 [Reference]
Expansion state	0.76 (0.71-0.81)	0.73 (0.67-0.80)	0.76 (0.70-0.83)

Abbreviation: aOR, adjusted odds ratio.

^a^ For 2017, n = 51 734 for health-related risk factors; n = 51 734 for insurance type; n = 12 608 for Medicare type; and n = 51 771 for Medicaid expansion.

^b^ For 2018, n = 40 452 for health-related risk factors; n = 40 452 for insurance type; n = 12 170 for Medicare type; and n = 40 467 for Medicaid expansion.

^c^ For 2019, n = 43 014 for health-related risk factors; n = 43 014 for insurance type; n = 12 876 for Medicare type; and n = 43 026 for Medicaid expansion.

^d^ Analyses of health-related risk factors are adjusted for age (15-39, 40-64, and >64 years); sex (male, female); region (Northeast, Midwest, South, or West); race and ethnicity (Hispanic, non-Hispanic Asian, non-Hispanic Black, non-Hispanic White, and other race and ethnicity); family income relative to poverty level (<100%, 100%-199%, 200%-299%, 300%-399%, and ≥400%); insurance coverage (private insurance without high deductible, private insurance with high deductible, Medicare, military, Medicaid, uninsured).

^e^ Insurance analyses are adjusted for sex; region; race/ethnicity; family income relative to poverty; hospitalized during year (yes, no); health status (excellent, very good, good, fair, poor); disability status (disabled, not-disabled); and age.

^f^ Medicare analyses are adjusted for sex; region; race and ethnicity; family income relative to poverty level; hospitalized during year (yes, no); health status (excellent, very good, good, fair, poor); and disability status (disabled, not disabled).

^g^ Medicaid expansion analyses are adjusted for same covariates as the insurance analyses, with region excluded because of colinearity.

The associations between health coverage and medical debt were also found in adjusted models. The uninsured had higher odds of having medical debt, as did those with high-deductible private insurance (compared with those with non–high deductible private insurance), whereas military and Medicaid coverage were associated with lower odds. Among Medicare beneficiaries, those with Medicare Advantage had the highest likelihood of medical debts whereas those with Medicare plus private coverage had the lowest. Residence in a Medicaid-expansion state was also associated with lower odds of medical indebtedness (eg, OR, 0.76; 95% CI, 0.70-0.83 in 2019).

In adjusted models, persons with medical debt had 2 to 3 times higher odds of food insecurity, being unable to pay rent or mortgage and utilities, and experiencing eviction or foreclosure (Table 3).

**Table 3.** Cross-sectional Association of Social Determinants of Health Outcomes With Medical Debt, Stratified by Year

Variable	Unadjusted, %^a^		Adjusted OR (95% CI)^b^
	Among persons with medical debt	Among persons without medical debt	
**2017**			
Unable to pay rent or mortgage	13.05	4.68	2.50 (2.22-2.81)
Unable to pay utility bills	19.44	6.50	2.86 (2.61-3.13)
Low or very low food security	23.82	8.49	2.71 (2.50-2.93))
Moved due to eviction or foreclosure	1.04	0.49	1.68 (1.20-2.36)
**2018**			
Unable to pay rent or mortgage	12.16	4.17	2.72 (2.30-3.21)
Unable to pay utility bills	18.10	6.04	2.95 (2.61-3.34)
Low or very low food security	23.49	8.18	2.76 (2.46-3.10)
Moved due to eviction or foreclosure	1.28	0.53	2.00 (1.35-2.96)
**2019**			
Unable to pay rent or mortgage	10.78	4.07	2.26 (1.97-2.61)
Unable to pay utility bills	16.47	5.84	2.52 (2.23-2.98)
Low or very low food security	19.82	7.29	2.51 (2.24-2.80)
Moved due to eviction or foreclosure	1.19	0.44	2.28 (1.56-3.35)

Abbreviation: OR, odds ratio.

^a^ For 2017, the number for analysis is 51 771; for 2018, 40 736; and for 2019, 43 177.

^b^ Adjusted for age (15-39, 40-64, and >64 years); sex (male, female); region (Northeast, Midwest, South, and West); race and ethnicity (Hispanic, non-Hispanic Asian, non-Hispanic Black, non-Hispanic White, and other race and ethnicity); family income relative to poverty level (<100%, 100%-199%, 200%-299%, 300%-399%, and ≥400%). For 2017, the number for analysis is 39 680; for 2018, 31 379; and for 2019, 35 859. Odds ratio greater than 1 indicates that persons with medical debt (vs those without debt) had a greater odds of experiencing the adverse social determinant of health.

### Prospective Results

Findings from prospective analyses (adjusted for income change and baseline characteristics) were congruent with the cross-sectional results. The odds of newly acquiring medical debt were elevated (in all cohorts) among those with coverage loss, increasing out-of-pocket spending and worsening health (Table 4). For example, becoming newly uninsured between 2017 and 2019 was associated with an OR, of 1.63; (95% CI, 1.23-2.14) for acquiring medical debt; becoming disabled with an OR of 2.42; (95% CI, 1.95-3.00); and experiencing hospitalization in 2019 but not 2017 with an OR, of 2.95; (95% CI, 2.40-3.62).

**Table 4.** Prospective Association of Change in Health and Medical Payment Factors and the Outcome of Newly Acquiring Medical Debt, Adjusted for Baseline Characteristics and Decrease in Income

Risk factor	New acquisition of medical debt, aOR (95% CI)^a^^,^^b^		
	Between 2017 and 2018	Between 2018 and 2019	Between 2017 and 2019
New hospitalization^c^	2.78 (2.30-3.35)	2.91 (2.29-3.71)	2.95 (2.40-3.62)
Newly disabled	2.48(2.02-3.04)	1.85 (1.39-2.47)	2.42 (1.95-3.00)
Newly uninsured	1.73 (1.33-2.24)	1.48 (1.05-2.11)	1.63 (1.23-2.14)
Out-of-pocket cost increase^d^	1.18 (1.12-1.24)	1.13 (1.06-1.22)	1.21 (1.15-1.26)

Abbreviation: aOR, adjusted odds ratio.

^a^ All models are adjusted for the following characteristics: region (Northeast, Midwest, South, and West); race and ethnicity (Hispanic, non-Hispanic Asian, non-Hispanic Black, non-Hispanic White, and other race and ethnicity); age (15-39, 40-64, ≥65 years); sex (male, female); baseline family income relative to poverty level (<100%, 100%-199%, 200%-299%, 300%-399%, and ≥400% of the federal poverty line); and decrease in family income category between the earlier and later years.

^b^ N = 25 130 for 2018 vs 2017; 16 723 for 2019 vs 2018; and 16 396 for 2019 vs 2017.

^c^ New hospitalization indicates no hospitalization in earlier year, but 1 or more hospitalizations in later year.

^d^ Out-of-pocket medical cost increase was modeled in continuous dollars; the odds ratio shown is per $2000 to facilitate interpretation.

The elevated odds of acquiring medical debt if newly disabled or hospitalized were also found after additional adjustment for being continuously insured during the period when debt was acquired, consistent with insurance offering some, but incomplete protection (eTable 3 in the Supplement). For instance, after such adjustment, the OR for acquiring medical debt between 2017 and 2019 among those newly hospitalized was 2.85 (95% CI, 2.32-3.49), and the comparable OR for those with new disability was 2.22 (95% CI, 1.79-2.75).

Conversely, those acquiring medical debts had consistently higher odds of worsening SDOH (Table 5). For instance, acquiring medical debt between 2017 and 2019 was associated with ORs of 2.20; (95% CI, 1.58-3.05) for becoming food insecure; 2.29; (95% CI, 1.73-3.03) for becoming unable to pay rent or mortgage; 2.37; (95% CI, 1.75-3.23) for becoming unable to pay utilities; and 2.95; (95% CI, 1.38-6.31) for eviction or foreclosure in 2019.

**Table 5.** Prospective Analyses of Associations Between the Acquisition of Medical Debt and Deterioration in Social Determinant of Health Outcomes, Adjusted for Baseline Characteristics and Decrease in Income

Outcome	Acquired medical debt during period, aOR (95% CI)^a^
**Less food secure**	
2018 vs 2017	1.70 (1.34-2.17)
2019 vs 2018	2.15 (1.59-2.91)
2019 vs 2017	2.20 (1.58-3.05)
**Less able to pay utilities**	
2018 vs 2017	2.12 (1.68-2.68)
2019 vs 2018	2.74 (1.99-3.79)
2019 vs 2017	2.37 (1.75-3.23)
**Less able to pay rent or mortgage**	
2018 vs 2017	2.29 (1.73-3.03)
2019 vs 2018	2.85 (2.05-3.96)
2019 vs 2017	2.07 (1.46-2.94)
**Evicted during year**	
2018 vs 2017	1.71 (0.90-3.24)
2019 vs 2018	3.07 (1.51-6.23)
2019 vs 2017	2.95 (1.38-6.31)

Abbreviation: aOR, adjusted odds ratio.

^a^ Odds ratio greater than 1 indicates that persons newly acquiring medical debt (vs others) had a greater odds of newly experiencing the adverse social determinant of health. N = 16 478 for 2019 vs 2017; 16 836 for 2019 vs 2018; 25 376 for 2018 vs 2017. All models are adjusted for the following characteristics: region (Northeast, Midwest, South, or West); race and ethnicity (Hispanic, non-Hispanic Asian, non-Hispanic Black, non-Hispanic White, and other race and ethnicity); age (15-39 years, 40-64 years, ≥65 years); sex (male, female); baseline income relative to poverty level (<100%, 100%-199%, 200%-299%, 300%-399%, and ≥400% of the federal poverty line); and decrease in family income category between the earlier and later year.

### Sensitivity Analyses

Compared with 2018 survey respondents who were resurveyed in subsequent years, those surveyed only in 2018 were younger, had lower income, and were healthier (characteristics associated with greater likelihood of moving), but had similar rates of medical debt (eTable 4 in the Supplement). Multivariable models carried out separately on those with and without longitudinal data yielded similar results (eTable 5 in the Supplement).

Analyses examining lagged effects yielded similar but slightly attenuated estimates to our main models; all ORs except 1 were significant (eTable 6 and eTable 7 in the Supplement).

## Discussion

One in 11 American men, 1 in 8 women, and nearly 1 in 5 households carry medical debt. US residents with middle-class or low incomes bear the brunt of this debt burden; only the highest-income and most educated segments of society are relatively spared.

As may be expected, our findings implicate poor and worsening health, and particularly hospitalization, as risk factors for medical debt. For those who became disabled or newly experienced hospitalization, health insurance offered only partial protection. Although the uninsured ran the greatest risk of medical debt, 2 increasingly widespread types of private coverage – high-deductible plans and Medicare Advantage – appeared to leave enrollees particularly exposed. The protective effect of military and Medicaid coverage – which carry minimal out-of-pocket costs – suggests the salience of comprehensive coverage, a point reinforced by our finding that Medicaid expansion was associated with lower rates of medical debt. Our counterintuitive findings regarding Medicare Advantage may reflect those plans’ benefit structures that offer low upfront costs but require high out-of-pocket payments for out-of-network care, prolonged hospitalizations, and some other services required by patients with serious illnesses.^11,12^

Our findings suggest that incurring medical debt leaves many unable to pay for utilities, and worsens housing and food security, key SDOH associated with adverse health outcomes,^13,14^ including frailty at birth.^15^ Hence, unaffordable medical bills may constitute an SDOH in their own right and contribute to a downward spiral of ill-health and financial precarity.

The prevalence of medical debt and beneficial outcomes of Medicaid expansion we observed accord with estimates from studies using credit reports.^7,8,16,17,18,19^ Our finding that persons with low income or middle income had similar rates of medical debt are consistent with those from a 2014 survey,^20^ but differs from a study that estimated incomes from debtors’ zipcodes.^7^ Surveys by the Kaiser Family Foundation^9^ and the Commonwealth Fund^1^ documented widespread problems paying medical bills and associated hardships, but included few specifics on medical debt. Similar to our analysis, they and others^21^ have found that medical bill problems were associated with medical need. Patients with cancer appear at especially high risk of financial toxicity^22^ from both medical bills and employment loss.^23^ Several studies indicate that medical debt decreases at age 65,^24,25^ in accord with our finding that Medicare coverage is somewhat protective. Those studies did not explore differences according to types of Medicare coverage. Surveys of debtors identified through court records suggest that medical issues contribute to many personal bankruptcies^26,27^ (and possibly to foreclosures),^28^ but could not assess populationwide medical indebtedness. To our knowledge, no previous studies have assessed medical debts and subsequent eviction.

### Limitations

This study has limitations. Observational data cannot prove causality or its direction; debts could deter outpatient care-seeking and thereby increase hospitalizations, whereas deteriorating SDOH could increase medical problems, use of care, and debts. Our conclusions from prospective analyses assume that medical events preceded the acquisition of debts, and that acquiring debts preceded worsening SDOH, but the timing of these events in a given year cannot be established in the SIPP. However, risk factors almost certainly preceded outcomes in our sensitivity analyses of lagged effects, suggesting that reverse-causation does not explain our findings.

The SIPP data are self-reported and hence subject to recall and other biases. Dollar amounts are often reported in round numbers and top-coding leads to underestimation of means. Medicare Advantage coverage was apparently underreported. We lacked detailed data on the components of out-of-pocket costs (eg, copayments) or specific insurance provisions that might increase such costs (eg, network restrictions). Although we controlled for income (and wealth) shocks, no data were available on increases in nonmedical spending, although our sensitivity analyses that controlled for changes in assets and in nonmedical debts addressed this point. Unmeasured factors, eg, variations in sick leave policies or the stringency of insurance regulation, could confound the association between Medicaid expansion and medical indebtedness.

Data collection difficulties in 2019 and 2020 compromised response rates.^29,30^ However, SIPP-provided weights account for nonresponse and Census Bureau analyses indicate that data for those years are generally reliable (T. Smith, PhD, US Census Bureau, written communication, December 10, 2021). Although small differences should be interpreted cautiously, the consistency of our findings across years is notable.

## Conclusions

Medical care inflicts debt on nearly one-fifth of US households, including many with seemingly good health insurance and middle-class incomes. Findings of this cohort study suggest that those debts appear to undermine housing and food security, with likely repercussions for debtors’ future health.

Hospitals and clinics might attenuate these problems by upgrading financial assistance programs, and forbearance in collecting debts. Clinicians’ efforts to screen for adverse SDOHs and make appropriate referrals may also be useful.^31^

However, clinicians’ efforts cannot substitute for policy change. Expanding Medicaid coverage nationwide may help reduce medical indebtedness. Eradicating medical debt would require implementing universal coverage that eliminates burdensome out-of-pocket costs.

## Notes

Supplement. **eMethods.** Insurance and Medicare Types **eTable 1.** Characteristics of Adults With and Without Medical Debt: 2017, 2018 and 2019 **eTable 2.** Share of US Adults Carrying Medical Debt, by Person-Level Characteristics, and Median Medical Debts Among Debtors, 2017, 2018 and 2019 **eTable 3.** Prospective Association of Acquisition of New Medical Debt (Outcome) with Newly Being Hospitalized or Newly Acquiring Disability, Adjusted for Baseline Characteristics and Change in Income, with Additional Adjustment for the Presence or Absence of Continuous Health Insurance Coverage in the Later Year(s) **eTable 4.** Characteristics of Adults in 2018 SIPP With and Without Data Available in Subsequent Years’ SIPPs **eTable 5.** Multivariate Odds Ratios in Cross Sectional Models of Predictors of Medical Debt and of Social Determinants of Health for Respondents to the 2018 SIPP, Stratified by Whether or Not They Also Provided Data in Subsequent SIPPs **eTable 6.** Lagged Analyses: Prospective Association of Change in Health and Medical Payment Factors 2017-2018 with the Outcome of Newly Acquiring Medical Debt between 2017 and 2019, Adjusted for Characteristics in 2017 and Fall in Income Between 2017 and 2019 **eTable 7.** Lagged Analyses: Prospective Association of the Acquisition of Medical Debt Between 2017 and 2018 With Worsening Social Determinants of Health Between 2017 and 2019, Adjusted for Characteristics in 2017 and Fall in Income Between 2017 and 2019
