# Supplementary material for: Prevalence and Risk Factors for Medical Debt and Subsequent Changes in Social Determinants of Health in the US
Source: JAMA Netw Open. 2022 Sep 16;5(9):e2231898. doi: 10.1001/jamanetworkopen.2022.31898 (PMC9482049; doi:10.1001/jamanetworkopen.2022.31898)
Supplement: Supplement. — eMethods. Insurance and Medicare Types eTable 1. Characteristics of Adults With and Without Medical Debt: 2017, 2018 and 2019 eTable 2. Share of US Adults Carrying Medical Debt, by Person-Level Characteristics, and Median Medical Debts Among Debtors, 2017, 2018 and 2019 eTable 3. Prospective Association of Acquisition of New Medical Debt (Outcome) with Newly Being Hospitalized or Newly Acquiring Disability, Adjusted for Baseline Characteristics and Change in Income, with Additional Adjustment for the Presence or Absence of Continuous Health Insurance Coverage in the Later Year(s) eTable 4. Characteristics of Adults in 2018 SIPP With and Without Data Available in Subsequent Years’ SIPPs eTable 5. Multivariate Odds Ratios in Cross Sectional Models of Predictors of Medical Debt and of Social Determinants of Health for Respondents to the 2018 SIPP, Stratified by Whether or Not They Also Provided Data in Subsequent SIPPs eTable 6. Lagged Analyses: Prospective Association of Change in Health and Medical Payment Factors 2017-2018 with the Outcome of Newly Acquiring Medical Debt between 2017 and 2019, Adjusted for Characteristics in 2017 and Fall in Income Between 2017 and 2019 eTable 7. Lagged Analyses: Prospective Association of the Acquisition of Medical Debt Between 2017 and 2018 With Worsening Social Determinants of Health Between 2017 and 2019, Adjusted for Characteristics in 2017 and Fall in Income Between 2017 and 2019 [file jamanetwopen-e2231898-s001.pdf]

Supplemental Online Content

Himmelstein DU, Dickman SL, McCormick D, Bor DH, Gaffney A, Woolhandler S. Prevalence and risk factors for medical debt and subsequent changes in social determinants of health in the US. *JAMA Netw Open.* 2022;5(9):e2231898. doi:10.1001/jamanetworkopen.2022.31898

eMethods. Insurance and Medicare Types

eTable 1. Characteristics of Adults With and Without Medical Debt: 2017, 2018 and 2019

eTable 2. Share of US Adults Carrying Medical Debt, by Person-Level Characteristics, and Median Medical Debts Among Debtors, 2017, 2018 and 2019

eTable 3. Prospective Association of Acquisition of New Medical Debt (Outcome) with Newly Being Hospitalized or Newly Acquiring Disability, Adjusted for Baseline Characteristics and Change in Income, with Additional Adjustment for the Presence or Absence of Continuous Health Insurance Coverage in the Later Year(s)

eTable 4. Characteristics of Adults in 2018 SIPP With and Without Data Available in Subsequent Years' SIPPs

eTable 5. Multivariate Odds Ratios in Cross Sectional Models of Predictors of Medical Debt and of Social Determinants of Health for Respondents to the 2018 SIPP, Stratified by Whether or Not They Also Provided Data in Subsequent SIPPs

eTable 6. Lagged Analyses: Prospective Association of Change in Health and Medical Payment Factors 2017-2018 with the Outcome of Newly Acquiring Medical Debt between 2017 and 2019, Adjusted for Characteristics in 2017 and Fall in Income Between 2017 and 2019

eTable 7. Lagged Analyses: Prospective Association of the Acquisition of Medical Debt Between 2017 and 2018 With Worsening Social Determinants of

© 2022 Himmelstein DU et al. *JAMA Network Open*.

Health Between 2017 and 2019, Adjusted for Characteristics in 2017 and Fall in Income Between 2017 and 2019

This supplemental material has been provided by the authors to give readers additional information about their work.

© 2022 Himmelstein DU et al. *JAMA Network Open*.

eMethods

Specification of Insurance Type

Persons can have multiple types of insurance simultaneously. Our analyses use the following hierarchy for classification of insurance into six mutually exclusive types. Persons with any private plan during December (the period when they were queried regarding debts) were categorized as "Privately insured, without high deductible." Those with private coverage who also said that their private plan had a high deductible were then recategorized as "High deductible private insurance". Next, those with any Medicare were recategorized as "Medicare", whether or not they also had private insurance. Then those with any military coverage were categorized as "Military" (whether or not they had private insurance or Medicare). Then those with Medicaid were categorized as "Medicaid" whether or not they had Medicare or any other insurance. Finally, persons who were uninsured were categorized as "uninsured" regardless of their coverage in other months. In prospective analyses, we categorized individuals as continuously insured vs. uninsured for one month or more.

Specification of Medicare Types

Among persons with Medicare at any time during the year, we created three categories. The first category was Medicare with any private insurance which we refer to as "Traditional Medicare + private". The second category was "Medicare Advantage" (also known as Medicare Part C or Medicare HMO). The third category "Traditional Medicare, no Private" included persons enrolled in Medicare with neither private insurance nor Medicare Advantage; this third category includes persons with Medicare + Medicaid (a group classified as having "Medicaid" in analyses using the insurance categories described above) .

© 2022 Himmelstein DU et al. *JAMA Network Open*.

eTable 1: Characteristics of Adults With and Without Medical Debt: 2017, 2018 and 2019

	Medical Debt 2019		Medical Debt 2018		Medical Debt 2017	
	Weighted % of Total With Debt (N = 4,434)	Weighted % of Total Without Debt (N = 38,743)	Weighted % of Total With Debt (N = 4,329)	Weighted % of Total Without Debt (N = 36,407)	Weighted % of Total With Debt (N = 6,005)	Weighted % of Total Without Debt (N = 45,766)
**Sex (%)**						
**Male**	41.0	49.3	40.8	49.3	41.9	49.2
**Female**	59.0	50.7	59.2	50.7	58.1	50.8
**Age category (%)**						
**15-39**	35,4	41.2	35.4	41.0	35.9	41.2
**40 - 64**	41.2	37.4	51.0	37.8	51.5	38.1
**65+**	23.4	21.4	13.6	21.2	12.7	20.7
**Race/ethnicity (%)**						
**White, non-Hispanic**	58.6	62.5	61.2	62.7	59.9	63.6
**Black, non-Hispanic**	20.1	11.0	16.5	11.4	18.2	11.0
**Asian, non-Hispanic**	2.7	6.6	2.5	6.3	2.3	6.3
**Other, non-Hispanic**	2.8	2.7	3.7	2.7	3.5	2.7
**Hispanic**	15.8	17.2	16.2	16.9	16.1	16.5
**Marital status (%)**						
**Married**	48.8	48.7	49.8	50.2	48.0	50.0
**Single, widowed, divorced**	51.2	51.3	50.2	49.8	52.0	50.0
**Income relative to poverty (%)**						
**<100**	12.0	10.1	12.1	10.8	14.2	12.1
**100-199**	21.3	14.1	23.5	15.1	21.5	14.8

© 2022 Himmelstein DU et al. *JAMA Network Open*.

**200-299**	20.4	14.1	21.8	15.0	20.7	14.7
**300-399**	14.8	13.1	15.4	13.2	14.8	13.0
**>=400**	31.6	48.5	27.3	45.8	28.8	45.4
**Education (%)**						
**< High school**	13.9	12.5	14.2	13.1	15.2	13.0
**High school**	35.8	33.3	36.2	33.4	37.1	34.0
**Some college**	40.4	33.7	40.6	33.7	39.6	33.6
**Graduate degree**	9.9	20.6	9.1	19.9	8.1	19.3
**Insurance (%)**						
**Private, not high deductible**	24.4	28.3	21.9	27.5	22.9	27.2
**Private, high deductible**	28.0	25.3	28.4	24.9	29.1	25.1
**Medicare (any type)**	14.6	17.1	15.1	17.3	15.3	17.7
**Medicaid**	14.9	13.9	17.4	15.5	15.7	14.8
**Military**	2.5	5.1	3.8	5.8	3.6	5.4
**Uninsured**	15.7	9.4	13.4	9.0	13.4	9.4
**Health Status (%)**						
**Excellent**	12.2	27.1	10.7	26.7	11.9	28.3
**Very good**	24.0	33.2	23.3	32.2	23.2	31.7
**Good**	33.9	26.2	31.8	26.9	33.0	26.1
**Fair**	20.9	10.0	23.9	10.6	21.8	10.2
**Poor**	9.0	3.5	10.4	3.7	10.1	3.6
**Medicaid Expansion State (%)**						
**Yes**	61.5	70.1	61.8	70.4	62.6	70.5
**No**	38.5	29.9	38.3	29.6	37.4	29.5
**Region**						
**Northeast**	**15.0**	**17.7**	14.1	18.1	14.0	18.2

© 2022 Himmelstein DU et al. *JAMA Network Open*.

**Midwest**	**22.8**	**20.5**	23.9	20.4	23.5	20.6
**South**	**46.3**	**37.0**	45.9	36.9	44.8	36.8
**West**	**15.7**	**24.8**	16.1	24.7	17.7	24.5

© 2022 Himmelstein DU et al. *JAMA Network Open*.

eTable 2: Share of US Adults Carrying Medical Debt, by Person-Level Characteristics, and Median Medical Debts Among Debtors, 2017, 2018 and 2019

Characteristic	Subgroup	% With Any Medical Debt			Median Amount of Medical Debt ($s)		
		2019	2018	2017	2019	2018	2017
**Sex**							
	Male	8.77	8.97	9.80	2000	2000	2000
	Female	11.84	12.20	12.76	1700	2000	2000
**Age**							
	15-39	9.03	9.31	10.02	2000	1570	2000
	40-64	13.39	13.84	14.72	2000	2000	2000
	65+	7.28	7.09	7.24	2000	1250	2000
**Race/ethnicity**							
	White, non-Hispanic	9.77	10.40	10.74	2000	2000	2000
	Black, non-Hispanic	17.39	14.73	17.44	1570	2000	1600
	Asian, non-Hispanic	4.54	4.47	4.54	1700	2000	1000
	Other, non-Hispanic	10.48	9.80	15.10	2000	2000	2000
	Hispanic	9.63	10.25	11.08	1500	2000	1500
**Marital status**							
	Married	10.37	10.56	10.93	2000	2000	2000
	Single, widowed, divorced	10.34	10.71	11.73	1500	2000	1800
**Income relative to poverty**							
	<100	12.02	11.78	13.05	2000	2000	2000
	100-199	14.84	15.56	15.66	1800	2000	2000
	200-299	14.28	14.71	15.24	1800	2000	2000
	300-399	11.50	12.15	12.69	1500	2000	2000
	>=400	6.99	6.62	7.49	1800	2000	1600

© 2022 Himmelstein DU et al. *JAMA Network Open*.

**Education**							
	< High school	13.55	14.00	15.73	2000	2000	2000
	High school	13.18	14.02	14.86	2000	2000	2000
	Some college	14.51	15.35	15.85	1800	2000	2000
	Graduate degree	6.36	6.41	6.28	1500	2000	1500
**Insurance**							
	Private (any type)	10.22	10.24	11.08	1800	2000	1500
	Private, not high deductible	8.72	8.65	9.89	1300	1500	1500
	Private, high deductible	11.82	11.94	12.37	2000	2000	2000
	Medicare (any type)	9.10	9.42	9.82	2000	1900	1700
	Medicaid	10.92	11.76	11.98	2000	1500	1500
	Military	7.18	7.35	5.93	1500	1850	1400
	Uninsured	14.17	15.05	16.49	2500	2000	2200
**Type of Medicare Coverage**							
	Medicare Part C	9.95	10.11	10.75	1300	1500	1500
	Traditional Medicare + private	7.52	7.42	7.40	2000	2000	1650
	Traditional Medicare, no private	12.60	12.86	13.71	2000	1500	2000
**Health Status**							
	Excellent	4.94	4.55	5.10	1500	1400	1500
	Very good	7.70	7.93	8.54	1500	1500	1500
	Good	13.03	12.38	13.91	1600	1800	1800
	Fair	19.43	21.16	21.42	2000	2200	2000
	Poor	23.21	24.94	26.13	4000	2800	3000
**Disability status**							
	Disabled	18.88	18.92	20.73	2000	2000	2000
	Not disabled	8.79	9.01	9.67	1500	2000	1600

© 2022 Himmelstein DU et al. *JAMA Network Open*.

**Hospitalized in past year**							
	Yes	21.82	22.82	23.57	3000	3000	3000
	No	9.11	9.23	9.89	1500	1700	1500
**Number of doctor visits in year**							
	0 or 1	7.72	7.56	7.95	1500	1800	1700
	2 or 3	8.71	8.69	9.89	1200	1500	1540
	4+	15.01	15.71	16.70	2000	2000	2000
**Number of Sick Days**							
	None	7.06	6.79	7.56	1500	1300	1500
	1 to 3	10,47	11.20	11.85	1500	2000	1500
	>3	20.68	21.42	21.39	2000	2500	2300
**Medicaid Expansion State**							
	Yes	9.20	9.45	10.19	1500	1800	1600
	No	12.96	13.34	13.93	2000	2000	2000
**Food Insecurity**							
	Secure	9.08	9.02	9.61	1800	2000	1900
	Moderately insecure	21.90	22.64	23.83	1700	2000	2000
	Very insecure	27.13	29.83	30.43	2000	2000	2000
**Unable to Pay Utilities**							
	Yes	24.58	26.28	27.64	2000	2000	2000
	No	9.29	9.40	9.92	1900	2000	1700
**Unable to Pay Mortgage/Rent**							

© 2022 Himmelstein DU et al. *JAMA Network Open*.

	Yes	23.42	25.77	26.25	2000	2000	2000
	No	9.70	9.84	10.44	2000	2000	1640
**Moved Because of Eviction/Foreclosure**							
	Yes	22.33	22.19	21.23	3000	3500	1500
	No	10.29	10.56	11.27	2000	2000	1800
**Region**							
	Northeast	8.90	10.51	8.95	1500	1500	1150
	Midwest	11.39	12.24	12.75	1800	2000	2000
	South	12.68	12.91	13.47	2000	2000	2000
	West	6.82	7.19	8.45	1500	1730	2000
**All Persons > 14**		**10.36**	**10.64**	**11.33**	1800	2000	2000

Note: Mean debts are not displayed because they are not comparable across years due to differences in top-coding: debts were top-coded at $273,000 in 2019; at $963,000 in 2018; and at $475,000 in 2017.

© 2022 Himmelstein DU et al. *JAMA Network Open*.

eTable 3: Prospective Association of Acquisition of New Medical Debt (Outcome) with Newly Being Hospitalized or Newly Acquiring Disability, Adjusted for Baseline Characteristics and Change in Income, with Additional Adjustment for the Presence or Absence of Continuous Health Insurance Coverage in the Later Year(s)

	**2018 vs. 2017 OR (95% CI) (N = 25,376)**	**2019 vs. 2018 OR (95% CI) (N = 16,836)**	**2019 vs. 2017 OR (95% CI) (N = 16,478)**
New hospitalization	2.61 (2.17-3.13)	2.96 (2.33-3.76)	2.85 (2.32-3.49)
Insured all of 2018	0.65 (0.53-0.78)	NA	0.78 (1.57-1.08)
Insured all of 2019	NA	0.69 (0.53-0.88)	0.74 (0.55-0.99)
Newly disabled	2.19 (1.80-2.67)	1.90 (1.42-2.53)	2.22 (1.79-2.75)
Insured all of 2018	0.66 (0.54-0.88)	NA	0.77 (0.56-1.07)
Insured all of 2019	NA	0.70 (0.54-0.89)	0.75 (0.56-1.00)

New hospitalization indicates no hospitalization in earlier year, and one or more hospitalizations in later year.

Newly disabled indicates not disabled in earlier year and disabled in later year.

All models are adjusted for the following characteristics: region (Northeast, Midwest, South, West); race/ethnicity (White non-Hispanic, Black non-Hispanic, Asian non-Hispanic, other race non-Hispanic, Hispanic); age (15-39, 40-64, 65+); sex; educational attainment (less than high school, high school, any college, graduate degree); baseline family income relative to poverty (<100, 100-199, 200-299. 300-399, 400+ percent of the federal poverty line); and change in income category.

© 2022 Himmelstein DU et al. *JAMA Network Open*.

eTable 4: Characteristics of Adults in 2018 SIPP With and Without Data Available in Subsequent Years' SIPPs

	Longitudinal Data Available (weighted % of Total)	No Longitudinal Data Available (weighted % of Total)
**Has medical debt (%)**	11.1	11.8
**Sex (%)**		
**Male**	48.3	49.5
**Female**	51.7	50.5
**Age category (%)**		
**15-39**	48.5	36.6
**40 - 64**	37.1	40.7
**64+**	14.4	22.7
**Race/ethnicity (%)**		
**White, non-Hispanic**	65.0	55.8
**Black, non-Hispanic**	10.7	13.7
**Asian, non-Hispanic**	5.6	5.7
**Other, non-Hispanic**	2.9	3.8
**Hispanic**	15.7	21.1
**Marital status (%)**		
**Married**	52.7	65.9
**Single, widowed, divorced**	47.3	34.1
**Income relative to poverty (%)**		
**<100**	11.5	19.0
**100-199**	15.5	17.1
**200-299**	15.2	15.0
**300-399**	13.1	12.6
**>=400**	44.8	36.4
**Education (%)**		
**< High school**	13.1	13.7
**High school**	33.8	35.9
**Some college**	33.7	35.8
**Graduate degree**	19.4	14.7
**Insurance (%)**		

© 2022 Himmelstein DU et al. *JAMA Network Open*.

**Any private**	64.8	61.9
**Any Medicare**	23.9	10.6
**Any Medicaid**	14.2	24.9
**Any military**	5.4	3.9
**Uninsured one month or more**	10.1	9.5
**Health Status (%)**		
**Excellent**	26.3	41.6
**Very good**	30.8	27.8
**Good**	26.8	20.5
**Fair**	11.8	7.3
**Poor**	4.4	2.7
**Region**		
**Northeast**	16.7	18.0
**Midwest**	21.0	20.8
**South**	37.3	38.7
**West**	25.0	22.6

© 2022 Himmelstein DU et al. *JAMA Network Open*.

eTable 5: Multivariate Odds Ratios in Cross Sectional Models of Predictors of Medical Debt and of Social Determinants of Health for Respondents to the 2018 SIPP, Stratified by Whether or Not They Also Provided Data in Subsequent SIPPs

	OR (95% CI) for individuals with > 1 year of data	OR (95% CI) for individuals with only 1 year of data
**Models with medical debt as outcome and health- or insurance-related characteristics as risk factors**		
Hospitalized during year	3.01 (2.72-3.33)	2.90 (2.53-3.31)
Disabled	2.71 (2.44-3.00)	2.65 (2.31-3.04)
Health status (vs. excellent)		
Poor	9.02 (7.47-10.90)	6.14 (4.87-7.74)
Fair	6.21 (5.31-7.26)	4.68 (3.79-5.53)
Good	3.42 (2.96-3.96)	2.68 (2.28-3.15)
Very good	1.90 (1.65-2.21)	1.37 (1.16-1.62)
Insurance (vs. private not-high deductible)		
Private, high deductible	1.44 (1.27-1.62)	1.12 (0.97-1.29)
Medicare	0.90 (0.76-1.08)	0.76 (0.60-0.98)
Military	0.41 (0.31-0.54)	0.43 (0.31-0.61)
Medicaid	0.57 (0.49-0.67)	0.61 (0.50-0.74)
Uninsured	1.40 (1.22-1.62)	1.19 (.99-1.42)
**Models with social determinants of health as outcome and medical debt as risk factor**		
Unable to pay rent/mortgage	2.42 (2.09-2.81)	2.63 (2.21-3.14)
Unable to pay utilities	2.90 (2.59-3.25)	2.79 (2.43-3.21)
Evicted	2.03 (1.34-3.07)	1.10 (0.58-2.10)
Food insecure	2.88 (2.61-3.18)	2.42 (2.11-2.77)

Note: As in the main cross sectional analyses, analyses of health-related predictor are adjusted for age (15-39, 40-64, >64 years); sex (male, female); region (Northeast, Midwest, South, West); Race/ethnicity (White non-Hispanic, Black non-Hispanic, Asian non-Hispanic, other race non-Hispanic, Hispanic); Family income relative to poverty (<100%, 100-199%, 200-299%, 300-399%, >=400%); Insurance coverage (Private insurance without high deductible, private insurance with high deductible, Medicare, military, Medicaid, uninsured). Analysis of insurance is adjusted for sex; region; race/ethnicity; family income relative to poverty; hospitalized during year (yes, no); health status (excellent, very good, good, fair, poor); and disability status (disabled, not disabled); and age (15-39, 40-64, >64 years).

Analyses of social determinants of health are adjusted for age (15-39, 40-64, >64 years); gender (male, female); region (Northeast, Midwest, South, West); Race/ethnicity (White non-Hispanic, Black non-Hispanic, Asian non-Hispanic, other race non-Hispanic, Hispanic); Family income relative to poverty (<100%, 100-199%, 200-299%, 300-399%, >=400%).

© 2022 Himmelstein DU et al. *JAMA Network Open*.

eTable 6: Lagged Analyses: Prospective Association of Change in Health and Medical Payment Factors 2017-2018 with the Outcome of Newly Acquiring Medical Debt between 2017 and 2019, Adjusted for Characteristics in 2017 and Fall in Income Between 2017 and 2019

**Predictor**	**New Acquisition of Medical Debt Between 2017 and 2019 Odds Ratio (95% CI)**
New hospitalization, 2018 vs. 2017	2.05 (1.58-2.67)
Newly disabled, 2018 vs. 2017	1.77 (1.29-2.42)
Newly uninsured, 2018 vs. 2017	1.88 (1.27-2.63)
Out-of-pocket cost rise, 2018 vs. 2017	1.12 (1.06-1.20)

New hospitalization indicates no hospitalization in 2017, but one or more hospitalizations in 2018.

Income fall indicates change between income categories (<100, 100-199, 200-299. 300-399, 400+ percent of the federal poverty line). Out-of-pocket medical cost rise odds ratio is continuous, with the OR shown for every $2000 rather per $1, to minimize the number of decimal places presented.

All models are adjusted for the following characteristics: region (Northeast, Midwest, South, West); race/ethnicity (White non-Hispanic, Black non-Hispanic, Asian non-Hispanic, other race non-Hispanic, Hispanic); age (15-39, 40-64, 65+); sex (male, female); income category in 2017 (<100, 100-199, 200-299. 300-399, 400+ percent of the federal poverty line); and change in income category between 2017 and 2019.as classified above.

© 2022 Himmelstein DU et al. *JAMA Network Open*.

Table S7: Lagged Analyses: Prospective Association of the Acquisition of Medical Debt Between 2017 and 2018 With Worsening Social Determinants of Health Between 2017 and 2019, Adjusted for Characteristics in 2017 and Fall in Income Between 2017 and 2019

**Outcome**	**Acquired medical debt during period Odds Ratio* (95% CI)**
Less food secure, 2019 vs. 2017	1.47 (1.01-2.13)
Less able to pay utilities, 2019 vs. 2017	1.91 (1.32-2.77)
Less able to pay rent or mortgage, 2019 vs. 2017	1.97 (1.30-2.97)
Evicted during 2019	1.74 (0.74-4.10)

* Odds ratio >1 indicates that persons newly acquiring medical debt (vs. others) had a greater odds of newly experiencing the adverse social determinant of health.
All models are adjusted for the following characteristics: region (Northeast, Midwest, South, West); race/ethnicity (White non-Hispanic, Black non-Hispanic, Asian non-Hispanic, other race non-Hispanic, Hispanic); age (15-39, 40-64, 65+); sex (male, female); baseline income category (<100, 100-199, 200-299. 300-399, 400+ percent of the federal poverty line); and fall in income category between 2017 and 2019.

© 2022 Himmelstein DU et al. *JAMA Network Open*.
